# Pilot Study of the Feasibility of a Worksite Plant-Based Diabetes Prevention Program

**DOI:** 10.1016/j.focus.2023.100064

**Published:** 2023-01-11

**Authors:** Zainab Almousa, Isobel R. Contento

**Affiliations:** 1 Department of Community Medicine and Behavioral Sciences, Faculty of Medicine, Kuwait University, Safat, Kuwait; 2 Teachers College, Columbia University, New York, New York

**Keywords:** Diabetes, prevention, plant-based, diet, lifestyle

## Abstract

**Introduction:**

This study aimed to explore the feasibility and acceptability of implementing a worksite diabetes prevention program―Beat Diabetes―on the basis of a plant-based diet and encouragement of physical activity.

**Methods:**

This was a mixed-methods pilot study using a 1-group pretest‒post-test design. The participants were university employees. A 13-week group-based intervention adapted from the Diabetes Prevention Program to emphasize a plant-based diet and on the basis of the Health Action Process Approach. Program implementation and participant acceptability were assessed through surveys, weekly documentation of the process, and individual interviews. Consumption of healthy and less healthy plant-based foods and animal-based foods, physical activity, and Health Action Process Approach‒based psychosocial determinants were measured through questionnaires before and after the program, as was blood glucose. Weights were measured weekly.

**Results:**

A total of 14 participants were enrolled, with 93% retention. Program implementation was feasible and highly acceptable to participants and also resulted in significant improvements in weight, healthy relative to less healthy plant-based and animal-based foods, vigorous physical activity, blood glucose, and psychosocial determinants of behavior change.

**Conclusions:**

On the basis of our pilot study, conducting a worksite plant-based diabetes prevention program was feasible and acceptable and elicited positive changes in physiologic, behavioral, and psychosocial variables related to the risk of diabetes.

## Highlights

• Diabetes affects millions of adults worldwide.
• The most effective way to control diabetes is through prevention.
• Studies exploring the links between plant-based diets and diabetes have seen a preventive effect.
• This study assesses the feasibility of a worksite plant-based diabetes prevention program.

## INTRODUCTION

The seventh leading cause of death in the U.S. is Type 2 diabetes.^1^^,^^2^ Diabetes affected 463 million adults (aged 20–79 years) worldwide in 2019 and is expected to increase to 700 million by 2045.^3^ Prevention is key, which on the basis of evidence from the Diabetes Prevention Program (DPP) and other studies is feasible with dietary change and physical activity.^4^^,^^5^ Studies report that smoking cessation, healthy eating, and maintaining a BMI <25 reduce diabetes risk by 90%.^6^

Studies exploring plant-based diets and diabetes report a preventative effect.^7^ The American Heart Association defines *plant based* as “A style of cooking and eating that emphasizes plant foods but is not strictly limited to them. Meat may be included but it's usually not the main feature of the meal.”^8^ A large prospective study of plant-based eating by Satija et al. found that those most adherent to a healthful plant-based diet had a 34% lower risk of developing diabetes than those least adherent.^9^ Two meta-analyses of controlled trials concluded that vegetarian and vegan diets can reduce HbA1c by an average of 0.3%–0.4% in people with Type 2 diabetes.^10^^,^^11^ Yet, no DPP studies have been found that are based on plant-based diets.

The primary aims of this study were to examine the feasibility of recruitment and participant retention, curriculum implementation, and acceptability to participants of a worksite plant-based DPP. The secondary aims were to examine changes in blood glucose, consumption of plant-based foods, psychosocial determinants of behavior change, physical activity, and weight over 13 weeks.

## METHODS

### Study Design

Beat Diabetes was a mixed-methods pilot study using a 1-group pretest‒post-test design. For its content, it used the Center for Disease Control and Prevention (CDC)’s Prevent T2 curriculum (https://www.cdc.gov/diabetes/prevention/resources/curriculum.html) that was modified to emphasize plant-based eating, illustrated with lunchtime meals. The physical activity sessions were unmodified from CDC's Prevent T2 modules (Appendix A, available online). Plant-based eating was emphasized by encouraging participants to eat more fruits and vegetables and explore plant sources of proteins (i.e., beans, legumes). Participants were not instructed to avoid animal protein but to limit portions.

The 13-week program was designed to be delivered in person, but owing to coronavirus disease 2019 (COVID-19), the first few sessions were delivered in person, and the remaining sessions were delivered on Zoom. The Teachers College, Columbia University IRB approved the study protocol.

### Study Sample

Participants had to be university employees, to be aged ≥18 years, to be nondiabetic, to not be pregnant, to be overweight/obese, and to have scored ≥5 on CDC diabetes risk test or have a diagnosis of prediabetes or previously had gestational diabetes. Methods of recruitment included gaining support from human resources and the employees’ union, presentations at employee meetings and the university health benefits fair, emails, flyers, and announcements on the college website. The theoretical framework for this program was the Health Action Process Approach (HAPA), often seen as an extension of social cognitive theory with an explicit focus on health behavior change and on a distinction between a motivational phase where psychosocial processes lead to a behavioral intention and a postintentional volition phase where behavior change processes lead to the actual health behavior.^12^^,^^13^ The phase structure of HAPA has been found predictive of behavior change^14^^,^^15^ and useful in many intervention studies, including a DPP.^16^^,^^17^ For provision of lunches, all onsite sessions were delivered in a space with a simple but well-equipped kitchen alcove. Weekly recipes illustrated the given week's behavior change goal. These were prepared by the research coordinator and 4 student volunteers with cooking experience. During virtual sessions, participants were encouraged to prepare the recipes.

### Measures

Feasibility was assessed by documenting the strategies used to recruit and retain participants. A checklist was completed weekly by program volunteers to assess the timely and complete delivery of lesson content and participant engagement, each scored 1–3 and then was converted to percentages, with >67% considered high.^18^ Two measures were used for participant acceptability and satisfaction: a Qualtrics survey with 33 open-/closed-ended questions to measure participant satisfaction and acceptability and individual Zoom interviews with 4 open-ended questions to capture their experience. Participants’ weights were measured weekly when sessions were in person using Tanita SC-331S scales (Tanita Corporation of America Inc., 2016). When sessions moved online, participants were asked to send their weekly weights using home scales. Participants who did not own a scale were sent one. Blood glucose measurements were made before and after the program using A1CNow SELF_CHECK. Before the program, the finger prick was done by the research coordinator, and after the program, each participant was sent an A1CNow SELFCHECK packet and provided with video instructions. The change in foods consumed was based on analysis of the 151-item Harvard semiquantitative food frequency questionnaire^19^ (https://regepi.bwh.harvard.edu/health/FFQ/files/2007%20GRID%20FFQ.pdf) administered before and after the intervention. Intakes were converted to servings per day and placed into 18 food groups, which were categorized into 3 groups as created by Satija et al. (2016), labeled as healthy plant foods, less healthy plant foods, and animal foods.^9^ The International Physical Activity Questionnaire (IPAQ)-short form, consisting of 7 questions, was used to assess physical activity over the past 7 days before and after.^20^ Psychosocial determinants of behavior were assessed before and after the intervention using an instrument based on the HAPA*’s* theoretical framework.^17^ The HAPA Psychosocial Determinants Questionnaire consists of 25 questions that assess perceived risk, outcome expectancies, action self-efficacy, behavioral intention, action self-efficacy, action planning, coping self-efficacy, and recovery self-efficacy directed at diet and exercise changes for weight loss as the behaviors of interest. All preprogram questionnaires were completed in person. After the program, participants were emailed or mailed a packet with a return envelope containing the food frequency questionnaire, IPAQ, and HAPA questionnaire. https://regepi.bwh.harvard.edu/health/FFQ/files/2007%20GRID%20FFQ.pdf

### Data Analysis

To assess program implementation, a quantitative analysis of the lesson completion and participant engagement checklists was conducted. To assess participants’ satisfaction and overall experience with the program, quantitative analyses of the Qualtrics surveys and qualitative analyses of the post-program interviews were conducted. No formal thematic analysis was completed. Common themes were identified in participants’ answers to the interview questions and reported. Only the research coordinator was involved in the analysis process.

Paired *t*-tests were used to examine differences in outcomes pre‒postintervention mean scores of all the quantitative measurements (SPSS, Version 27.0, IBM Corp, 2020): dietary intake, IPAQ, HAPA questionnaire, and weight. Pearson correlations were used to determine the associations between HAPA variables, weight loss, physical activity, and diet.

## RESULTS

Most participants were recruited at the health fair where they took the diabetes risk test and learned more about the program. Others were from visits to offices and emails. Ultimately, 41 signed up, and 18 met the inclusion criteria of a diagnosis of prediabetes and/or a score above 5 on the diabetes risk test. Of these, 14 enrolled. Table 1 shows that the participants were diverse, except that all had some college education.

**Table 1:** Sample Demographic Characteristics (N=14)

Variables	% (number of participants)
Age, years	
<40	7 (1)
40–49	29 (4)
50–59	43 (6)
≥60	21 (3)
Sex	
Male	29 (4)
Female	71 (10)
Other	
Hispanic or Latino	
Yes	36 (5)
No	64 (9)
Racial/ethnic group^a^	
American Indian or Alaska Native	7 (1)
Hispanic, Latino, or Spanish origin	36 (5)
White	21 (3)
Asian	0
Middle Eastern or North African	7 (1)
Black or African American	43 (6)
Native Hawaiian or other Pacific Islander	0
Other	7 (1)
BMI	
Overweight (25–29.99)	7 (1)
Obese Class 1 (30–34.99)	36 (5)
Obese Class 2 (35–39.99)	29 (4)
Obese Class 3 (≥40)	29 (4)
Highest degree or level of school completed	
Less than high-school degree	0
High-school degree or equivalent (e.g., GED)	0
Some college, no degree	21 (3)
Bachelor's degree (e.g., BA, BS)	36 (5)
Graduate degree (e.g., MA, MS, PhD, EdD)	43 (6)

a For racial‒ethnic group, participants were instructed to check all that apply, so numbers do not add up to 14.

Motivators to join included health concerns or perceived risk, outcome expectations or perceived benefits, and perceived barriers (Table 2). Perceived risk predominated, with 43% stating that diabetes ran in their family. Perceived benefits in terms of weight and diabetes prevention were other health concerns.

**Table 2:** Participants’ Reasons for Joining the Program

Reasons for joining the program	% (number of participants) N=14
Health concerns (perceived risk)	
High blood pressure	7 (1)
Overweight	21 (3)
Diabetes runs in the family	43 (6)
Diagnosed as prediabetic	21 (3)
Fear of developing diabetes	7 (1)
Lost family members to diabetes	7 (1)
Outcome expectations (perceived benefits)	
Increase knowledge of health	14 (2)
A better understanding of portion size	21 (3)
Increase nutritional literacy	7 (1)
Learn skills and habits to prevent diabetes	7 (1)
Lower blood sugar	7 (1)
Improve blood pressure	7 (1)
Lose weight	43 (6)
Prevent diabetes	21 (3)
Improve health and longevity	14 (2)
Improve eating habits	21 (3)
Make better food decisions	7 (1)
Reduce time spent thinking about food	7 (1)
Establish a healthy relationship with food	7 (1)
Curb cravings	7 (1)
Less snacking	7 (1)
Get support for a healthy lifestyle	7 (1)
Increase physical activity	7 (1)
Staying healthy for children/grandchildren	14 (2)
Feel empowered to take control of your health	7 (1)
Outcome expectations (perceived barriers)	
Does not enjoy the taste of healthy food	7 (1)

Of the 14 participants enrolled, 13 (93%) completed the full 13-week program. One participant dropped out in Week 8 owing to a reported lack of time. It was feasible to provide educational lunches within the lunch hour. Meals included plant-based main dishes, vegetable sides, and fruits. Preparation was ∼2 hours. Cost was about $6/person (in 2021 U.S. dollars).

With regard to lesson completion and participant engagement, average scores for lesson completion for in-person sessions ranged from 91.25% to 100%, and for online lessons, average scores ranged from 84.5% to 100%, both representing high implementation. Participant engagement was high in person and medium to high online.

In terms of participant satisfaction, Qualtrics’ survey results showed participants’ positive responses to program components. All the 13 participants who completed the program considered the program very or extremely useful. They reported that visual demonstrations were extremely useful and that the information presented was very or extremely useful, especially information on reading labels and recipes. All the 13 participants reported being extremely satisfied with the program.

Participant experience was assessed through interviews. When describing their journey, 5 of the 13 participants said that they felt anxious after learning that they were at risk for diabetes, but after the program, they felt that their risk was modifiable. Others described not knowing the link between diet and diabetes risk and how the program gave them tools to take control of their health. Many felt that the Beat Diabetes team was supportive and created a safe nonjudgmental environment. Some mentioned that other participants’ support was helpful.

Reported participant successes included changing eating habits, portion control, weight loss, and more exercise. Contributors to success were weekly sessions, healthy lunches, group support, and knowledge and skills. Some challenges included finding time to exercise, reducing sweets, and lacking motivation.

In terms of outcome measures, all 14 participants completed the HbA1c pretest: 9 had normal HbA1c levels, 1 had prediabetes, and 4 had values in the diabetic range. One participant had a previous diagnosis of prediabetes. Only 11 participants completed the HbA1c post-test. Of the 9 individuals with normal initial values, 7 completed the post-test and remained in the normal range. The individual with prediabetes remained in the prediabetic range. Three of the 4 individuals who started with diabetic values completed the post-test. The individual with confirmed diabetes lost 3.89% of initial body weight and had a drop in HbA1c from 8.7% to 8.1%. The second individual lost 4.63% of initial body weight, and HbA1c dropped from 6.6% (diabetic) to 5.8% (prediabetic). The third individual lost 0.11% of initial body weight and remained in the diabetic range.

Weight change from before to after the program was significant. Of the 13 program completers, 11 (85%) lost weight, and 2 gained weight. The pre-test and post-test mean weights were 220.12 ± 38.82 lbs and 214.67 ± 37.08 lbs, respectively. The mean weight loss was statistically significant at 5.45 ± 6.14 lbs (2.48% of initial body weight) (*p*=0.01). The average drop in BMI was 0.92 points. The participant who dropped out at Week 8 had attended 5 sessions and lost 2.6 lbs, but his data were not included in the analyses.

In terms of the change in consumption of healthy and less healthy plant-based and animal foods, diet quality improved. Average consumption of all food groups within the healthy plant-based category increased, except tea/coffee. The mean consumption of vegetables (*p*=0.037) and fruits (*p*=0.055) increased. For less healthy plant-based foods, mean consumption decreased for every group. The decrease in consumption of potatoes and sweets/desserts was significant (*p*=0.042 and *p*=0.029, respectively). Average consumption of foods under the animal food category also decreased, except for fish and seafood (which increased) and animal fat (which was unchanged). The decrease in miscellaneous animal foods was significant (*p*=0.035). The mean for teaspoons (tsps) of sugar per day dropped to 0.81 tsp but was not significant. However, 6 of the 7 participants who reported adding sugar initially reduced their intake, and their average decreased from 2.21 tsps to 1.5 tsps.

The IPAQ asks about vigorous and moderate physical activity, walking, and sitting time. Owing to COVID-19 and many working from home, participants’ physical activity patterns were involuntarily affected. On the basis of pre- and post-IPAQ assessments, walking and moderate physical activity decreased but not significantly. For vigorous physical activity, initially, only 2 of the 13 participants reported engaging in vigorous physical activity, but in the post-IPAQ assessment, 7 of the 13 participants (53%) were engaging in vigorous physical activity. The increase was significant (*p*=0.044).

HAPA psychosocial determinants’ scores all changed except for recovery self-efficacy. Perceived risk decreased. Outcome expectancies, which ask questions such as “If I lose weight…” followed by potential outcomes (e.g., “…I will have more energy”) increased. Behavioral intention and action and coping self-efficacy increased. The frequency of action planning increased (*p*=0.048). Frequency of planning was correlated with vigorous physical activity (*r*=0.57) (*p*=0.04).

In terms of associations between weight loss, diet, and exercise, there was a positive association (*r*=0.64) between decreased consumption of sweets/desserts and weight loss (*p*=0.018). Participants with the greatest weight loss had the greatest reduction in consumption of sweets/desserts. Refined grains had a moderate association with weight loss (*r*=0.67) (*p*=0.012). Reductions in meat consumption were also correlated with weight loss (*r*=0.57) (*p*=0.044). Reductions in miscellaneous animal products were strongly correlated with weight loss (*r*=0.77) (*p*=0.002).

## DISCUSSION

Implementation was feasible among participants of diverse racial/ethnic backgrounds. Significant changes in diet, physical activity, weight loss, and psychosocial determinants of behavior change were observed. Although recruitment was difficult, similar to most studies,^21^ maintenance was high (93%), despite going virtual midway through.

Introducing a plant-based diet was a major attraction of the program and not a barrier to enrollment. Participants of diverse cultural backgrounds were enthusiastic about it. Providing plant-based lunches was reported as helpful by participants. Considering the increased intake of more healthy plant foods and decreased intake of less healthy plant foods and animal foods and that decreases in intakes of sweets/desserts, refined grains, and meat were significantly associated with weight loss suggests that a plant-based diet approach may be useful in DPPs. Although many DPPs are offered during the lunch hour, none have been found that offer a meal as an educational instrument. Future DPPs might consider adding a food component to facilitate dietary change. Use of theory created behaviorally focused lessons: HAPA's emphasis on a motivation phase followed by an action phase and action plans was useful. Although the sample size was too small to analyze associations between improvements in HAPA determinants and behavior change, larger studies should do so.

The program's success can also be attributed to the educational techniques used, key among them being facilitated dialogue. Beat Diabetes provided a level of comfort that allowed participants to openly discuss their health with colleagues and anticipate feedback. Creating safe environments is a pillar of facilitated group discussion.^22^, ^23^, ^24^, ^25^, ^26^ This occurs when individuals feel respected and sense their opinions being valued.

Another contributor to the program's success was social support. When participants were asked who provided them with the most support, almost 70% said the Beat Diabetes team. Mohr et al. (2011) created a supportive accountability model, stating that receiving support from a trustworthy coach increases adherence. Adherence also improves with clear expectations. The program had clear behavior change goals, and participants were involved in action planning. Weight loss is important for diabetes prevention; however, expectations set for participants should be based on processes or behaviors (e.g., eating fewer processed snacks) as opposed to outcomes such as weight loss, which lower adherence and increase distress.^27^

### Limitations

Limitations included a small sample size, no control group, and self-selection. For the qualitative data, threats to validity included researcher bias and reactivity or influence of the researcher on participants and respondent bias. Triangulation was used to address these threats.

## CONCLUSIONS

Conducting a worksite plant-based DPP was feasible and acceptable and resulted in positive changes in physiologic, behavioral, and psychosocial variables related to the risk of diabetes. Future larger controlled trials can further explore plant-based diets in diabetes prevention.

## Appendix Supplementary materials

Image, application 1

## Footnotes

Supplementary material associated with this article can be found in the online version at doi:10.1016/j.focus.2023.100064.
