# Supplementary material for: Pilot Study of the Feasibility of a Worksite Plant-Based Diabetes Prevention Program
Source: AJPM Focus. 2023 Jan 11;2(2):100064. doi: 10.1016/j.focus.2023.100064 (PMC10546524; doi:10.1016/j.focus.2023.100064)
Supplement: Supplementary file 1 [file mmc1.docx]

**Appendix A**

**Appendix Table 1**. **Beat Diabetes** **Session Titles, Behavior Change Goals, and Corresponding Recipes**

| **Session Title** | **Behavior Change Goal** | **Recipes** |
| --- | --- | --- |
| **In-Person** | | |
| Session **1:** An Introduction to Beat Diabetes | • Be more mindful of food intake and stopping when you feel full.  • Engage in 10-15 minutes of physical activity each day | - Crisp Black Bean Tacos with Feta and Cabbage Slaw - Quinoa Salad with Corn, Tomatoes, Avocado, and Lime |
| Session **2:** Understanding Diabetes and How to Protect our Bodies | • Replace processed snacks high in refined sugar with healthier items such as fruits or vegetables with healthy dips. | - Sandwich with Beetroot Hummus - Open-Faced Avocado Tuna Sandwich - Green Salad with Apples, Cranberries and Pepitas - Apple Ring and Peanut Butter Sandwiches |
| Session **3:** Energy Balance and Weight Loss Tips and Tricks | • Make your meals resemble the Healthy Eating Plate | - Baked Tofu with Peanut Sauce - 20-Minute Vegan Brown Rice Salad with Greens |
| Session **4:** Greens and Grains: A Little Greener, a Little Grainier, and a lot Healthier | • Make two “Small Changes” from the Healthy Eating Plate, which are:  - Choose a fruit and/or vegetable at every meal AND  - Choose 2 whole grain alternatives to replace 2 refined grain products that they currently consume. | - Shrimp and Zucchini Barley Risotto - Mediterranean Quinoa Salad |
| Session **5:** Revamping Your Meals: Exploring More Protein Options | • Choose one meal a day to be vegetarian  • Try out Meatless Mondays | - Sweet Potato & Black Bean Chili - Whole-Grain Cornbread - Roasted Broccoli |
| Session **6:** Get Active to Prevent T2 ^a^ | • Be a little more active  • Try out different activities to find one you enjoy | - Vegan Mushroom Walnut Bolognese - Roasted Cauliflower Salad with Creamy Honey Mustard Vinaigrette |
| **Online** | | |
| Session **7:** Track Your Activity ^a^ | • Identify the purpose of tracking activity  • Describe how to track their activity | - Tex Mex Chicken and Zucchini - One Pan Broccoli Quinoa Skillet with Parmesan and White Beans |
| Session **8:** How Sweet It Is: Choosing Better Beverages for Health at Every Sip | • Commit to drinking water most of the time and limiting intake of sugar sweetened beverages. | - Vegan Jambalaya - Chili Lime Roasted Cauliflower - One-Ingredient Banana Ice-cream |
| Session **9:** Get More Active ^a^ | • Identify some ways to get more active.  • Explain how to track more details about their fitness Commit to planning their meals ahead of time and food shopping accordingly. | - Garlic Honey Ginger Glazed Salmon with Broccoli - Super-carb Loaded Sweet Potatoes |
| Session **10:** My Plate, My Cart: Shop Smart for Better Health | • Commit to planning meals ahead of time and food shopping accordingly. | - Salmon Patty Recipe - Quick Vegetable Stir-Fry |
| Session **11:** Manage Stress ^a^ | • Identify some ways to reduce stress and ways to cope with stress. | - Chickpea Salad Sandwich - Citrus Avocado Salad - Peanut Butter Bliss Balls |
| Session **12:** Eating Out: How to Make Healthy Choices | • Commit to making healthy decisions that support weight loss goals when eating out or ordering in. | - Caramelized Onion and Artichoke Flatbread - Shaved Brussel Sprouts Salad |
| Session **13:** Stay Motivated to Prevent T2^a^ | • Reflect on how far they have come since they started this program and set goals for the next 6 months. | - Spinach Artichoke Lasagna - Roasted Corn Tomato Summer Salad with Lemon-Basil Yogurt Dressing |

1. Sessions were unmodified from those in CDC Prevent T2 Curriculum, but lunches and recipes were still provided during these sessions.

**Appendix Table 2. Comparison between Beat Diabetes and CDC’s Prevent T2 Curriculum**

| **Beat Diabetes’ Curriculum (13 Sessions)** | **CDC’s Prevent T2 Curriculum-First 6 months of Program (16 Sessions)^a^** |
| --- | --- |
| Session **1:** An Introduction to Beat Diabetes | Introduction to the Program |
| Session **2:** Understanding Diabetes and How to Protect our Bodies | Eat Well to Prevent T2 |
| Session **3:** Energy Balance and Weight Loss Tips and Tricks | Track Your Food |
| Session **4:** Greens and Grains: A Little Greener, a Little Grainier, and a lot Healthier | Burn More Calories than you Take In |
| Session **5:** Revamping Your Meals: Exploring More Protein Options | Find Time for Fitness |
| Session **6:** Get Active to Prevent T2 | Get Active to Prevent T2 |
| Session **7:** Track Your Activity | Track Your Activity |
| Session **8:** How Sweet It Is: Choosing Better Beverages for Health at Every Sip | Cope with Triggers |
| Session **9:** Get More Active | Get More Active |
| Session **10:** My Plate, My Cart: Shop Smart for Better Health | Shop and Cook to Prevent T2 |
| Session **11:** Manage Stress | Manage Stress |
| Session **12:** Eating Out: How to Make Healthy Choices | Eat Well Away from Home |
| Session **13:** Stay Motivated to Prevent T2 | Stay Motivated to Prevent T2 |
|  | Keep Your Heart Healthy |
|  | Take Charge of Your Thoughts |
|  | Get Support |

1. With the exception of the first session in the CDC’s Prevent T2 curriculum, which has to be delivered first, the remaining sessions can be delivered in any order over the span of 6 months. <https://www.cdc.gov/diabetes/prevention/resources/curriculum.html>
